# Induced pluripotent stem cells in Alzheimer’s disease: applications for disease modeling and cell-replacement therapy

**DOI:** 10.1186/s13024-016-0106-3

**Published:** 2016-05-17

**Authors:** Juan Yang, Song Li, Xi-Biao He, Cheng Cheng, Weidong Le

**Affiliations:** Center for Translational Research on Neurological Diseases, the First Affiliated Hospital, Dalian Medical University, 116021 Dalian, China; Key Laboratory of Stem Cell Biology, Institute of Health Sciences, Shanghai Institutes for Biological Sciences, Chinese Academy of Sciences/Shanghai Jiao Tong University School of Medicine, 200031 Shanghai, China; Collaborative Innovation Center for Brain Science, the First Affiliated Hospital, Dalian Medical University, 116011 Dalian, China; Shanghai University of Medicine and Health Sciences, Shanghai, 201318 China

**Keywords:** Alzheimer’s disease, Cell-replacement therapy, Disease modeling, Drugs screening, Induced pluripotent stem cells

## Abstract

Alzheimer's disease (AD) is the most common cause of dementia in those over the age of 65. While a numerous of disease-causing genes and risk factors have been identified, the exact etiological mechanisms of AD are not yet completely understood, due to the inability to test theoretical hypotheses on non-postmortem and patient-specific research systems. The use of recently developed and optimized induced pluripotent stem cells (iPSCs) technology may provide a promising platform to create reliable models, not only for better understanding the etiopathological process of AD, but also for efficient anti-AD drugs screening. More importantly, human-sourced iPSCs may also provide a beneficial tool for cell-replacement therapy against AD. Although considerable progress has been achieved, a number of key challenges still require to be addressed in iPSCs research, including the identification of robust disease phenotypes in AD modeling and the clinical availabilities of iPSCs-based cell-replacement therapy in human. In this review, we highlight recent progresses of iPSCs research and discuss the translational challenges of AD patients-derived iPSCs in disease modeling and cell-replacement therapy.

## Background

Alzheimer’s disease (AD) is the most common form of dementia that begins with short-term memory deficits and culminates in total loss of cognition and executive functions. The formations of amyloid-β (Aβ) plaques and intracellular neurofibrillary tangles (NFTs), two important pathological hallmarks of AD, have been linked to the synapse loss, neuronal degeneration and subsequent dramatic brain atrophy of AD patients [1, 2]. Although these clinical features and pathological profiles of AD have been well documented and various animal models containing AD-related genetic backgrounds have been developed, the etiological mechanisms for AD are still far from being fully understood and effective therapeutic strategies for AD are still urgently needed.

Dozens of drugs and therapeutic strategies attempting to slow or halt neuronal loss and cognitive deficiency of AD are being investigated around the world [3]. However, only five pharmacological agents have been approved for clinical AD treatment by the Food and Drug Administration, including cholinesterase inhibitors tacrine, donepezil, galantamine, rivastigmine and N-methyl-D-aspartate (NMDA) receptor antagonist memantine [4]. Unfortunately, all these currently available pharmacological therapeutics only relieve symptoms without affecting the major pathological characteristics of AD. Moreover, the effectiveness of these agents varies from person to person as evidenced by a moderate efficiency in no more than 20 % of patients and tolerance, noncompliance and side-effects in more than 60 % of treated patients [5].

Several theoretical hypotheses have been raised for elucidating the pathological mechanisms of AD, including amyloid-cascade hypothesis [6], tau hypothesis [7], mitochondrial cascade hypothesis [8], oxidative stress hypothesis [9] and neuroinflammation hypothesis [10]. Among them, amyloid-cascade hypothesis is widely accepted as the centerpiece of AD pathology, in which Aβ is recognized as the initiating factor in AD. Previous studies have reported that Aβ plaque deposition, an early critical event of AD, triggers the downstream features of AD including tangles formation, oxidative stress, neuroinflammation and neuronal loss. Several new candidates targeting Aβ have been tested in clinical trials in past years. Unfortunately, all of these therapeutic candidates failed to improve the cognitive and functional ability of AD patients, yet caused serious side-effects [11, 12]. Therefore, a better understanding of the upstream mechanisms of AD pathology is urgently required for the discovery of novel disease-modifying therapeutic strategies.

**Fig. 1:**
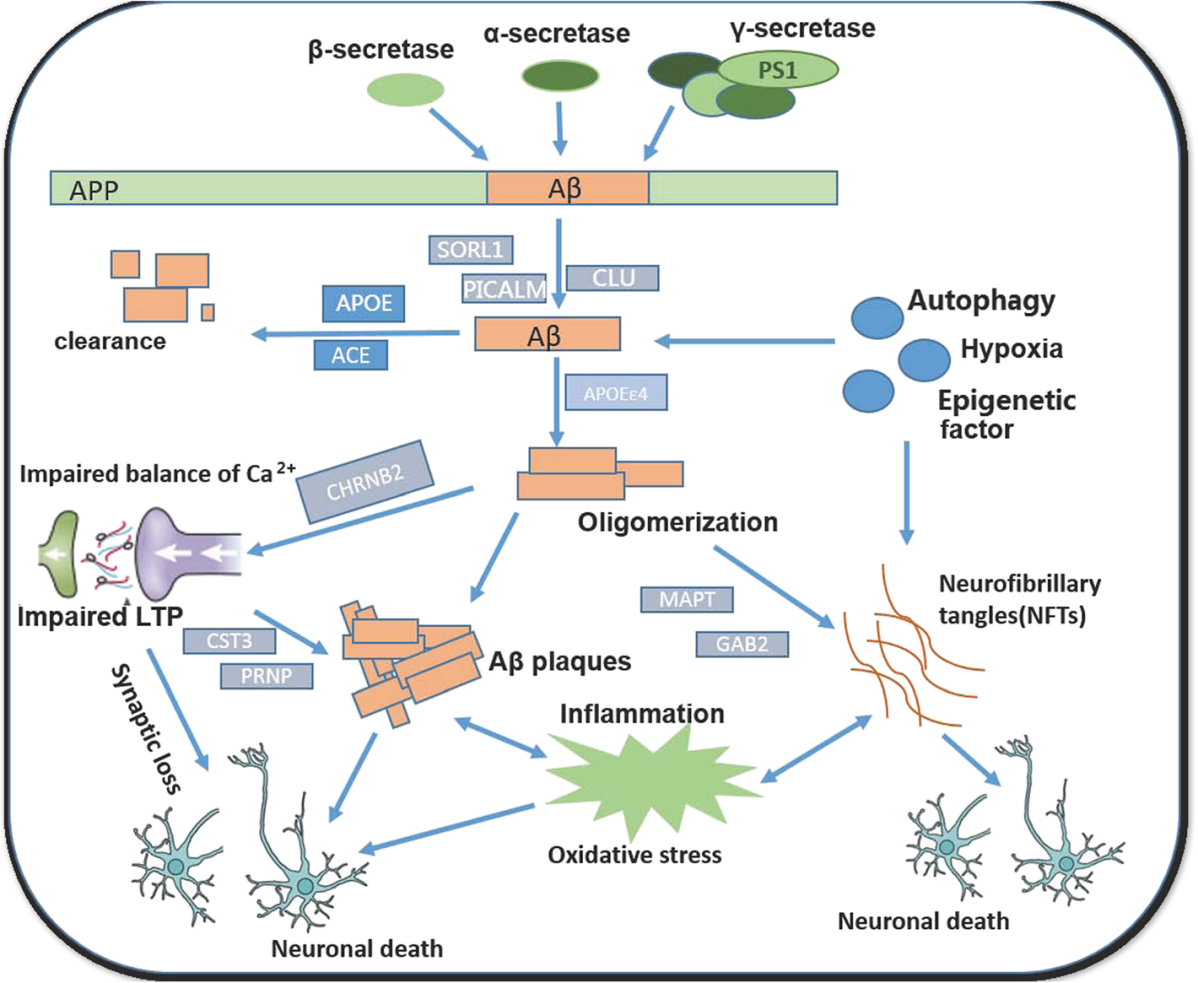
Schematic diagram of causative or risk factors for AD pathogenesis

Till now, at least 3 causal genes and 22 risk genes have been identified to be involved in the pathogenesis of AD, including amyloid precursor protein (APP), presenilin-1/2 (PSEN1/2) [5, 13], apolipoprotein E (APOE) [14], ABCA7, BIN1, CASS4, CD33, CD2AP, CELF1, CLU, CR1, DSG2, EPHA1, FERMT2, HLA-DRB5-DBR1, INPP5D, MS4A, MEF2C, NME8, PICALM, PTK2B, SORL1, SLC24H4-RIN3, and ZCWPW1 [15]. However, genetic factors could only partially explain the risk of AD. The notion widely accepted to date is that the onset of AD is most likely the consequence of complicated interactions of multiple genetic and non-genetic factors (Fig. 1) [16]. Several non-genetic risk factors have been proposed, including aging, cerebro-cardiovascular diseases, metabolic disorders, traumatic brain injury, sleep disorders, chronic hypoxia and environmental toxins [3, 17–23]. However, the mechanisms underlying them still remain largely unknown, due to the lack of non-postmortem and AD patient-specific research models.

The identification of AD causal genes resulted in the generation of more than 150 transgenic AD mouse models, by over-expressing one or more mutated genes such as human APP, PSEN1/2, or Tau [24]. These transgenic models have greatly advanced the understanding of AD pathogenesis and promoted the findings of novel therapeutic targets and strategies for AD treatment. However, none of these transgenic models can reflect all pathogenic and clinical features of AD. Different combinations and extents of gene mutations led to a great variety of AD phenotypes [25, 26]. Although Aβ plaques and cognitive impairment were observed in almost all animal models, NFTs could only be generated by lines over-expressing human tau protein. Cholinergic neuronal loss can be observed in several lines, but massive neuronal loss in brain is rarely observed in these animal models. Based on these, in the current stage transgenic animal models cannot fully recapitulate the progress of human AD. In addition, the transgenic animals carrying autosomal dominant familial AD (FAD) genes may have limitations in modeling sporadic AD (SAD) [27, 28]. Therefore, more representative models are needed for facilitating the fundamental AD research and exploring more efficient therapeutic strategies for AD treatment.

**Fig. 2:**
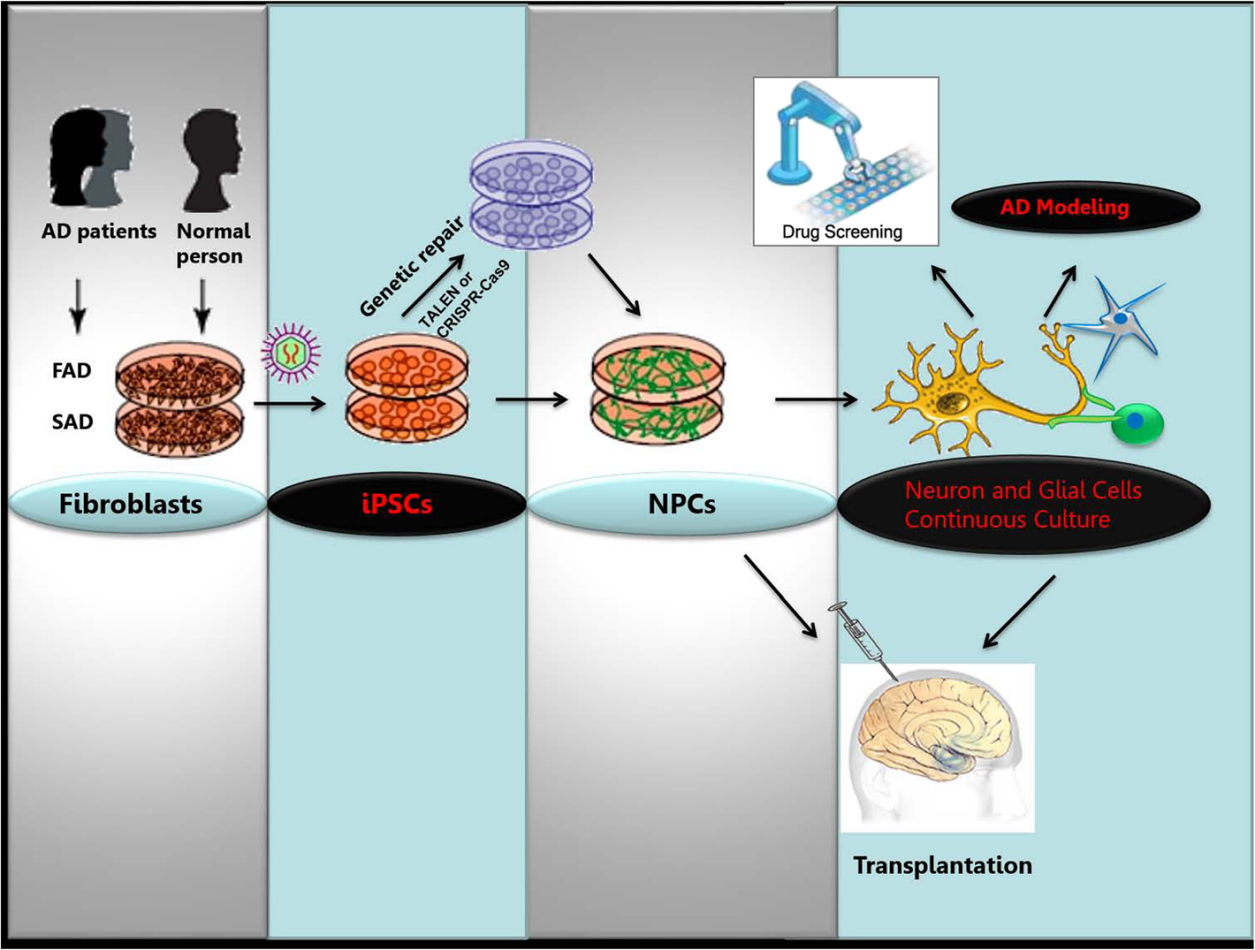
Potential applications of iPSCs technology in AD modeling, drugs screening and cell-replacement therapy

The recently developed and optimized induced pluripotent stem cells (iPSCs) technology may provide an appealing access to overcome these challenges in AD research. iPSCs can be generated from somatic cells by using several key transcription factors for pluripotency. iPSCs are in general identical to embryonic stem cells (ESCs) with the ability to self-renew unlimitedly and differentiate into all cell types [29–31]. Additionally, human iPSCs derived from patients’own somatic cells may serve as a sufficient cell source for clinical transplantation therapy (also termed as cell-replacement therapy), which can efficiently prevent immunologic rejection and the ethical issues raised by the use of ESCs [32, 33]. More importantly, human iPSCs derived from either FAD or SAD patients’ somatic cells contain a patient-specific pathogenic background, therefore, can provide a promising avenue for AD modeling, which bridges the gap between animal models and clinical testing. This strategy is extremely useful for the mechanism understanding of AD pathogenesis, clinical identifying of therapeutic targets and drug screening of the novel therapeutic candidates against AD (Fig. 2). In this review, we update the recent progress of iPSCs research, and discuss its potential applications, as well as the major challenges and future directions in disease modeling and cell-replacement therapy of AD.

## Progress of iPSCs research

In 2006, Yamanaka and colleagues found mouse fibroblast cells can be reprogrammed into iPSCs by using 4 transcription factors including Octamer-binding transcription factor 4 (Oct4), sex determining region Y-box 2 (Sox2), Kruppel-like factor 4 and cMyc [30]. In 2007, this technology was successfully applied in human somatic cells [29]. Additionally, a combination of Oct4, Sox2, NANOG and LIN28 has also been demonstrated to induce iPSCs from human somatic cells [29, 31]. After the first discovery of mouse or human iPSCs, considerable efforts have been made to develop or optimize this technology, including reprogramming cells by using fewer or newly defined factors and more efficient delivery systems.

Oct4 inhibits the expression of differentiation-related genes in ESCs [34–36] and has been reported to be sufficient to direct the reprogramming of somatic cells into iPSCs [37, 38]. Moreover, Shu et al. have found that lineage specifiers, which act as pluripotency rivals to suppress ESCs identity, can induce mouse fibroblasts into iPSCs [39]. Lineage specifiers that are involved in mesendodermal specification (such as GATA3, GATA6, and SOX7) and ectodermal specification (such as SOX1, SOX3, and GMNN) can synergistically induce pluripotency in the absence of Oct4 and Sox2, suggesting a “seesaw effect” of the lineage specifiers in cell reprogramming. Additionally, more and more novel reprogramming factors have been identified as pluripotency-associated factors or maternal factors, such as PR domain-containing 14, Sall4, Esrrb, Utf1, Tet1, Tet2, Gli-similar protein 1 and inhibitors of differentiation 3 [40–48].

Manipulations of microRNAs (miRs), such as miR-291-3p, miR-294 and miR-295, have been reported to increase the efficiency of reprogramming without the presence of cMyc [49]. Anokye-Danso and colleagues have also found that miR-302-367 can replace traditional reprogramming factors to reprogram mouse and human somatic cells into iPSCs with higher efficiency [50].

Besides the utilization of pluripotency transcription factors, the induction of iPSCs has been extended to involve various kinds of donor cells, including fibroblasts, glias, neural progenitors, human keratinocytes, liver and stomach cells, pancreatic-β cells, mature B lymphocytes, human amniotic fluid-derived cells, as well as cells in blood and urine [51–56].

Additionally, the delivering strategies for inducing iPSCs have been improved. The retroviral or lentiviral vectors were routinely used to integrate the reprogramming genes into the host genome to induce iPSCs [30, 57]. Viral delivery system are efficient and reproducible in reprogramming, however, the random integration of transgenes into genome increases the risk of tumor formation and may cause mortality in chimeric and progeny mice derived from iPSCs [58]. Transfection of linear DNA by polycistronic vectors using liposomes or electroporation can avoid viral integration, but the reprogramming efficiency of these methods is much lower than viral delivery. The piggyBac transposon delivery system is less risky because the integration of non-viral constructsis more stable than virus vectors [59]. Furthermore, many viral integration-free systems for iPSCs generation have been established, such as adenovirus, Sendai virus, minicircle vector, episomal vectors, and direct protein delivery [60–65]. Importantly, Hou et al. have reported that small-molecules, such as VC6T plus FSK, can replace “master genes” Sox2 and Oct4 to induce cell reprogramming [65, 66]. This chemical reprogramming strategy is relatively simple and less time-consuming, and has promising potential in generating functional desirable cell types for clinical applications.

## Application of iPSCs in AD modeling

The rapid development of iPSCs technology promotes the application of iPSCs in the research of neurodegenerative diseases. Since 2008, over 50 literatures have been published to demonstrate the neurodegenerative diseases modeling by using iPSCs, majorly generated from familial patients but a few from sporadic patients [32]. Among them, several research groups have reported the usage of iPSCs in AD modeling, which provide proof-of-principle for modeling patient-specific AD pathology by using iPSCs and recapitulate several pathological features of AD in vitro (Table 1).

**Table 1:** iPSCs-based AD modeling

Cell type	AD models	Phenotypes	Significance	Ref
FAD-iPSCs	Fibroblasts of FAD patient with mutations in PS1 and PS2	FAD-iPSCs-derived neurons have increased Aβ42 secretion; responds to γ-secretase inhibitors and modulators	Recapitulating the molecular pathogenesis of mutant PS; identification and validation of candidate drugs	[67]
FAD- and SAD-iPSCs	Fibroblasts of FAD patient with mutations in APP; sporadic AD	AD-iPSCs-derived neurons exhibited higher levels of Aβ, pTau, and active GSK-3β; β-secretase inhibitors caused significant reductions in pTau and active GSK-3β levels	The first SAD iPSC model; Demonstrating the direct relationship between APP processing in GSK-3β activation and tau phosphorylation	[68]
AD-iPSCs	Fibroblasts from AD patient	AD-specific iPSCs lines	Exploring AD pathologies; screening new drugs and therapeutic regimens	[70]
AD-iPSCs	Fibroblast of Down syndrome patients	Neurons generated from Down syndrome patients-iPSCs developed AD pathologies	Recapitulating AD pathogenic process including Aβ42 and hyperphosphorylated tau	[71]
FAD-iPSCs	PSEN1 mutant fibroblasts	produced greater ratios of Aβ42 to Aβ40; 14 genes differentially-regulated	Identify novel candidate genes during AD pathology	[72]
FAD-iPSCs	Human dermal fibroblasts	Aβ oligomers accumulation; elevated endoplasmic reticulum (ER) and oxidative stress;	Illustrating how patient-specific iPSCs can be useful for analyzing AD pathogenesis and evaluating drugs.	[73]
SAD-iPSCs				
FAD-iPSCs	Human fibroblast carrying APP mutation (V717I)	Increased APP expression and levels of APPsβ, Aβ; increased Aβ42 and Aβ38; increase in levels of total and phosphorylated Tau	Demonstrating the direct casual relationship between elevated Aβ and hyperphosphorylated tau using Aβ neutralizing antibodies, for the first time;	[74]
			Providing a model system for testing therapeutic strategies in the cell types most relevant to disease processes	

Yagi et al. firstly generated iPSCs from AD patients carrying familial mutations in *PSEN1* (A246E) and *PSEN2* (N141I) and reported that these FAD-derived iPSCs had an increased Aβ_42_ production and an elevated ratio of Aβ_42_/Aβ_40_ [67]. Then, Israel et al. generated iPSCs lines from two SAD patients (named as sAD1/sAD2) and two FAD patients with a duplication of APP (APP^Dp^) [68]. They found that neurons derived from APP^Dp^-iPSCs line and sAD2-iPSCs line have significantly higher levels of Aβ_40_, increased phosphorylation of tau protein (at Thr 231) together with an elevated level of active glycogen synthase kinase-3β (aGSK-3β). Additionally, neurons from those AD-derived iPSCs (AD-iPSCs) accumulated large RAB5-positive early endosomes, which is consistent with the findings from the neurons of AD patients [69]. More interestingly, treatment of the neurons with β-secretase inhibitors (BSI), but not γ-secretase inhibitors, could significantly reduce the levels of phospho-tau (Thr 231) and aGSK-3β, while γ-secretase inhibitors only reduced the level of Aβ_40_, suggesting that APP proteolytic processing, but not Aβ_40_, had a direct relationship with GSK-3β activation and tau phosphorylation in human neurons. Consistent with these findings, Jang et al. and Shi et al. have also generated neurons from iPSCs derived from primary fibroblast of AD patients [70, 71] and found these cells could recapitulate AD pathogenic process such as Aβ_42_ and hyperphosphorylated tau and could be used for screening new drugs and therapeutic regimens.

Sproul et al. also have found a higher Aβ_42_/Aβ_40_ ratio in the neural progenitor cells (NPCs) derived from AD-iPSCs harboring *PSEN1* A246E or M146L mutations [72]. Moreover, they identified 14 genes differentially-regulated in PSEN1 NPCs molecular profiling. Among these genes, *GFRA3*, *ISL1*, *DLX1*, *SEMA3B*, and *ERBB3* showed differential expression in late onset AD/Intermediate AD brains.

Kondo et al. generated seven AD-iPSCs lines, including three lines from a patient carrying APP E693 deletion (APP E693d), two lines from a patient harboring APP V717L mutation (APP V717L), and two lines from a SAD patient [73]. The authors found that Aβ oligomers accumulated in neurons derived from APP E693d-iPSCs and in neurons and astrocytes derived from one of the two SAD-iPSCs lines, which could be reduced by BSI. Furthermore, they found that the stress responses in the AD neural cells were alleviated by BSI and docosahexaenoic acid treatment. This study illustrates the possible application of patient-specific iPSCs for screening anti-AD drugs and classifying AD patients.

Muratore et al. generated four iPSCs lines from two FAD patients carrying APP V717I mutation and differentiated them into neurons expressing forebrain neuron marker [74]. Both β- and γ-secretase cleavage of APP were affected by this mutation. Elevated β-secretase cleavage of APP led to an increased level of both sAPPβ and Aβ, while the alteration of the initial cleavage site of γ-secretase resulted in an increased Aβ_42_ and Aβ_38_. Moreover, they found that the levels of total and phosphorylated tau were increased in neurons derived from AD patient. Furthermore, Aβ-specific antibodies could reverse the phenotype of increased total tau in AD-iPSCs derived neurons. These findings indicate that the tau-related changes are relevant to Aβ phenotype and the increased tau might be a consequence of Aβ generation, which is consistent with the amyloid-cascade hypothesis of AD.

Furthermore, forebrain cholinergic neuron (FBCN) loss is directly relevant to the memory and cognition deficits in AD. Therefore, generation of FBCNs from AD patient-specific iPSCs is crucial for disease modeling in vitro and for the development of novel AD therapies. Based on this, Duan et al. have recently reported that FBCNs derived from SAD-iPSCs showed typical AD biochemical features as evidenced by an increased Aβ_42_/Aβ_40_ ratio and a higher susceptibility to glutamate-mediated cell death [75].

Down syndrome (DS) patients with early-onset dementia share similar neurodegenerative features with those of AD. Chang et al. have recently found that accumulated amyloid deposits, tau protein hyperphosphorylation and tau intracellular redistribution emerged rapidly in DS-iPSCs-derived neurons within 45 days but not in normal ESCs-derived neurons, suggesting DS-iPSC-derived neural cells can serve as an ideal cellular model of DS and AD and have potential for high-throughput screening of therapeutic candidates [76].

## Challenges and future perspectives of iPSCs in AD disease modeling

The above mentioned research findings reveal several pathological features of AD, including Aβ generation and tau-phosphorylation, in AD-iPSCs derived from FAD and SAD patients, indicating the potential application of iPSCs technology in AD modeling and drug screening. However, there are still several concerns regarding AD-iPSCs models.

First of all, although iPSCs could serve as a promising avenue for disease modeling, the molecular principles for this technique, particularly in human cells, still remain poorly understood due to donor-to-donor variability and intercellular heterogeneity. In the study from Israel et al., only one of the two SAD-iPSCs lines has the same phenotypes as the APP^Dp^-iPSCs line. Similarly, Kondo et al. have also found that seven AD-iPSCs lines with APP E693 deletion or APP V717L mutation or SAD did not always recapitulate the same phenotypes [73]. These inconsistencies might suggest that although there are similar mechanisms underlying the pathogenesis of FAD or SAD, a larger sample size is still required to investigate the comprehensive and robust heterogeneity of AD phenotypes in future study. In addition, although AD patient-derived human neurons have shown elevated levels of toxic Aβ species and phosphorylated tau, they also could not recapitulate Aβ plaques or tau tangles. One of the possible reasons for this might be the lack of a proper cell culture system, which mimics the in vivo condition and provides a better microenvironment to achieve enough pathogenic Aβ accumulation, as observed in human AD patients and transgenic AD mouse models. Recently, Kim et al. have found that robust Aβ and tau pathologies could be achieved in a 3-dimension human neural cell culture models [77]. Although this model was based on genetically engineered human neural stem cell line, not iPSCs, it may provide a promising tool for human AD iPSCs models.

Secondly, the neural cells differentiated from iPSCs displayed AD pathological hallmarks in less than two months, suggesting that disease-related cells may be more susceptible to display the hallmarks of disease in cell culture dishes than in patients’ brains. However, there also remains the question that whether the one or two cell types differentiated from iPSCs can represent all complicate phenotypes of AD. In addition, the cells exhibited the pathological changes of AD, but synapse loss or neuronal degeneration was rarely observed. It has been reported that AD is a complex disease that affects both neuronal and glial activities and glia participates in Aβ clearance and inflammation in AD [78]. Therefore, further studies of glial cells derived from AD-iPSCs can facilitate the discovery of the exact mechanisms underling AD pathogenesis.

Thirdly, in these studies of AD-iPSCs, iPSCs derived from healthy individuals or family members were used as controls, which may have totally different genetic background from the AD-iPSCs. This kind of controls is not the most appropriate approach in disease modeling assays. Hence, isogenic iPSCs derived from AD-iPSCs, in which the mutations have been corrected to wild-type, are the most appropriate controls. Nowadays, isogenic, mutation-corrected control cell lines generated by transcription activator-like effector nucleases (TALENs) or clustered regularly interspaced short palindromic repeats (CRISPR)/CRISPR-associated protein (Cas) mediated genomic editing technology has been applied in human diseases, such as ALS, and tumors [79, 80].

Overall, AD-iPSC lines from more AD patients and further studies using both AD-iPSC-derived neuron and glial cells and isogenic controls can provide tools to discover more accurate features and mechanisms of AD.

## iPSCs-based cell replacement therapy for AD: the mechanisms and rationales

iPSCs have great potential in cell transplantation therapy of neurodegenerative diseases including AD. Transplantable neural progenitors or neurons can be generated from ESCs and iPSCs [81, 82]. NPCs derived from ESCs or iPSCs can be further differentiated into neurons, astrocytes or oligodendrocytes, which provide promising aspect for cell-replacement therapy of various neurodegenerative disorders including AD.

The dysfunction of neurogenesis has been found in AD mouse models, indicating that the worsened imbalance between neurogenesis and neuronal loss may contribute to the pathogenesis of AD [83]. It is generally accepted that adult neurogenesis primarily occurs in two sites important for learning and memory, the subventricular zone (SVZ) of the lateral ventricles and the subgranular zone (SGZ) of the dentate gyrus (DG) of the hippocampus. Wang et al. have reported that AD transgenic mice harboring PSEN1P117L show a decreased survival of NPCs, leading to a reduced production of new neurons. In addition, the reduced adult neurogenesis in DG has been suggested to be correlated with an impaired contextual fear conditioning in mouse [84–87]. In APP V717F mice, an age-dependent decrease of cell proliferation in SGZ of DG was also observed [88]. Research reports suggest that neurogenesis was significantly enhanced as a self-repairing mechanism to compensate for the early onset of neurodegeneration; however, the survival of newly generated neurons was impaired following neurodegeneration progression [89]. Interestingly, although the cellular composition and morphological organization of the SVZ in human and non-human primates differ from those of rodents [90, 91], the proliferation and migration of NPCs in the SVZ of young APP transgenic mice have also been reported to be greatly decreased [92], suggesting that Aβ plaques might be involved in the impaired neurogenesis in AD mouse model. However, it has been reported that the decreased NPCs and neuroblasts as wells as severely impaired proliferation and differentiation of NPCs occurred preceding the onset of amyloid deposition and memory impairment in 2-month-old APPswe/PSEN1ΔE9 mice or triple transgenic mice carrying APPswe, PSEN1-M146V and tau-P301L mutations [93]. These findings suggest that progressive neuronal loss and impaired neurogenesis may be important pathological events of AD.

Previous studies have found that transplanted NSCs differentiate into mature cell types and improve cognitive ability of AD animal models via various mechanisms, with or without the involvement of Aβ or tau pathologies. For instance, NSCs have been reported to rescue cognitive functions and promote synaptogenesis without altering Aβ or tau pathologies in AD mouse models [94–96]. Moreover, NSCs have also been found to attenuate the expression of proinflammatory cytokines and neuronal loss in AD model [97]. In contrast to these research reports, Lee et al. have revealed that human NSCs transplantation reduced tau phosphorylation, down-regulated BACE1 expression and Aβ production via Akt/GSK3β signaling, and decreased the expression of inflammatory mediators through deactivation of microglia [98]. As a primary source of NSCs, iPSCs, especially human-derived iPSCs, may provide a promising strategy for cell-replacement therapy of AD.

It is likely that the transplantation of NSCs can provide not only a direct cell-replacing strategy for AD therapy, but also can be used as vehicles for the delivery of potential therapeutic agents, including neprilysin, insulin-degrading enzyme, plasmin and cathepsin B, to reverse AD pathologies [99, 100]. It is suggested that the future NSCs- or iPSCs-based cell therapy in AD should focus on such indirect mechanisms [101–103].

## iPSCs-based cell replacement therapy for AD: hints from other CNS disorders

iPSCs have great potential as cell source in cell transplantation therapy of neurodegenerative diseases [104, 105]. One of the main purposes of iPSCs-based cell replacement therapy in AD and other neurodegenerative diseases is to produce new neurons to replace those lost or function-deficient cells during disease progression or to produce glial cells to protect neurons from ongoing degeneration. Data from in vivo studies suggest that iPSCs-derived neurospheres were able to survive and differentiate into neurons, astrocytes, and oligodendrocytes, after transplantation into injured mouse spinal cord [106]. In addition, previous research report has also indicated that after transplantation into the fetal mouse brain, NPCs derived from mouse iPSCs migrate into various brain regions and differentiate into glia and neurons [107], and thus are capable to integrate into preexisting functional neuronal circuitries in the CNS or compensate the degenerative neurons. Electrophysiological experiments and morphological observations demonstrate that the grafted neurons display normal neuronal activity and are functionally integrated into the host brain. All these findings support the notion that iPSCs- or iPSCs derivatives-based cell replacement therapy could be efficiently used to treat neurodegenerative diseases. Indeed, several lines of studies have tested this idea in experimental models of Parkinson’s disease (PD) and amyotrophic lateral sclerosis (ALS).

It has been reported that transplanted neural stem cells in parkinsonian rat striatum can release and reuptake dopamine and alleviate PD symptoms [108]. Human iPSCs-derived neurons can improve the functional defects of rotational asymmetry in PD rat model after transplantation [109]. Autologous iPSCs-derived dopamine neurons can provide long-term functional recovery in monkey model of PD [84]. In addition, glial-rich neural progenitors derived from human iPSCs can improve lifespan of ALS mice after being transplanted into the lumbar spinal cord [85].

As for AD, Fujiwara et al. have reported that the spatial memory of AD mice was improved significantly after grafted with human iPSCs-derived neural progenitors [110]. In addition, Huang’s group has found that after transplantation of the embryonic medial ganglionic eminence (MGE)-derived interneuron progenitors into the hippocampal hilus of aged apoE4-KI mice, the transplanted cells developed into mature interneurons, and the neurons functionally integrated into the hippocampal circuitry and rescued learning and memory [111]. Moreover, Nicholas et al. found that MGE-like progenitors derived from both ESCs and iPSCs could be induced to differentiate into GABAergic interneurons and displayed mature physiological properties in mouse brain up to 7 months post-transplantation [112]. These research findings indicate that transplanted human iPSCs-derived cells can functionally rescue pathological changes in the brains of AD patients. All these preclinical progresses in the investigation of cell transplantation in neurodegenerative diseases may provide proof-of-concept for the final clinical translation of iPSCs in therapy of AD.

Although great achievements have been made for iPSCs-based cell replacement therapy, one major challenge is teratoma formation. It has been demonstrated in the mouse system that iPSCs-derived chimeras frequently develop tumors, which should be carefully evaluated after transplantation before clinical application. Takahashi et al. calculated that approximately 20 % of the mice derived from iPSCs develop tumors [113]. Transgene reactivation and incomplete reprogramming are considered as the primary causes of tumorigenesis [114]. In order to reduce the risk of tumor formation that limits the clinic application of iPSCs, many studies have provided strategies. Wang et al. have introduced a mifepristone-regulated caspase-1 expression system to selectively eliminate tumor cells derived from undifferentiated ESCs but not differentiated dopamine neurons [115]. Cui et al. have identified that the suppression of canonical wingless-type MMTV integration site family (WNT) signaling pathway can reduce the tumorigenicity and substantially improved retinal integration of ESCs-derived retinal progenitor cells transplanted in mice [116]. In addition, Nori et al. have recently suggested that integration-free iPSCs should be chosen to avoid tumorigenesis [117]. Overall, these progresses of iPSCs research can provide new avenue for the clinical translation of cell transplantation therapy against various neurodegenerative diseases including AD.

## Conclusion

AD patient-specific iPSCs-derived neurons or glia cells can recapture not only familial, but also sporadic form of AD. Moreover, iPSCs provide unique platforms to detect the early-disease phenotypes during neurogenesis or neurodegeneration which may point towards underlying pathogenic mechanisms of AD. In addition, isogenic iPSCs of AD-iPSCs can be obtained by TALEN- or CRISPR/Cas-mediated genetic repairing technology. Genetically repaired AD-iPSCs can serve as more appropriate control cells for disease modeling and cells transplantation. Altogether, even though there are several challenges in the clinical usage of iPSCs technology, the recent promising achievements in this field will contribute significantly for exploring molecular mechanisms of AD and promoting clinical AD therapy.

## Abbreviations

BSI: β-secretase inhibitor
aGSK-3β: active glycogen synthase kinase-3β
AD: Alzheimer’s disease
Aβ: Amyloid-β (Aβ)
APP: Amyloid precursor protein
APOE: Apolipoprotein E
CRISPR: Clustered regularly interspaced short palindromic repeats
Cas: CRISPR-associated protein
DG: Dentate gyrus
DS: Down syndrome
APP^Dp^: Duplication of APP
ESCs: Embryonic stem cells
FAD: Familial AD
FBCN: Forebrain cholinergic neuron
iPSCs: Induced pluripotent stem cells
miRs: microRNAs
NMDA: N-methyl-D-aspartate
NPCs: Neural progenitor cells
NFTs: Neurofibrillary tangles
Oct4: Octamer-binding transcription factor 4
PSEN1/2: Presenilin-1/2
Sox: Sex determining region Y-box
SAD: Sporadic AD
TALENs: Transcription activator-like effector nucleases
SGZ: Subgranular zone
SVZ: Subventricular zone
